# A New Method of Using the Catheter, and Instantaneous Cure of Strictures

**Published:** 1855-12

**Authors:** 


A New Method of Using the Catheter, and Instantaneous Cure of Strictures.—M. Maisonneuve communicated on the 14th of May, 1855, to the Academy of Sciences at Paris, a memoir, in which he describes his method of catheterism, which may briefly be sketched as follows:

The author, being struck by the advantages of passing a tube over a conductor in the treatment of stricture, (as practiced by Mr. Wakely in this country,) proposes, when the stricture is very narrow, to introduce a filiform bougie into the bladder, to screw a metallic instrument to the free end of it, and to push on that instrument along the canal, making the bougie recede into the bladder, in the cavity of which it is expected to bend upon itself. The instrument used may be either of those which have been proposed to dilate strictures or to cut through them. The author thinks, however, that bad coarctations should be divided from before backwards, and he operates in the following manner: When the bougie has reached the bladder, a grooved urethrotome is pushed onwards through as many strictures as the canal may present, the bougie bending upon itself in the bladder. Along the groove, a rod, furnished with a sharp blade at its extremity, is pushed on, and made to cut the urethral obstacles. When this is done, the grooved urethrotome is withdrawn, and a second introduced, to the extremity of which a hidden blade is attached. As soon as the instrument is fairly in the canal, the blade is protruded by means of a spring; and on withdrawing this urethrotome caché, the strictures are again fully divided. The peculiarity of the method is, that the author considers that the freedom of the canal after these incisions would be prevented by leaving an instrument on the raw surfaces; he thinks that the part should be allowed to heal without any catheter, and maintains the cure is instantaneous, as the patient is immediately able to pass urine in a good stream. The Academy of Sciences have appointed a committee to report upon this method, M. Civiale being one of the members.—*London Lancet.*

